# Further contributions to the aleocharine fauna of the Yukon Territory, Canada (Coleoptera, Staphylinidae)

**DOI:** 10.3897/zookeys.186.2674

**Published:** 2012-04-26

**Authors:** Jan Klimaszewski, Benoit Godin, Caroline Bourdon

**Affiliations:** 1 Natural Resources Canada, Canadian Forest Service, Laurentian Forestry Centre, 1055 du P.E.P.S., P.O. Box 10380, Stn. Sainte-Foy, Québec, Quebec, Canada G1V 4C7; 2 Environment Canada, 91782 Alaska Highway, Whitehorse, Yukon, Canada Y1A 5B7

**Keywords:** Canada, Coleoptera, Staphylinidae, Aleocharinae, taxonomy, Yukon

## Abstract

The aleocharine beetles of the Yukon Territory, Canada are reviewed based on material studied since the most recent survey of the territory in 2008. The present contribution recognizes a fauna of 125 species, of which 9 are new to science, 20 represent new territorial records and one represents a new Canadian record. Seventeen species are considered Holarctic, 6 introduced, and 2 species are of undetermined status (Holarctic or adventive). The Yukon fauna is classified in 32 genera and 8 tribes. The new species are: 1) *Acrotona horwoodae* Klimaszewski & Godin, **sp. n.**; 2) *Atheta (Microdota) microelytrata* Klimaszewski & Godin, **sp. n.**; 3) *Atheta (Microdota) riparia* Klimaszewski & Godin, **sp. n.**; 4) *Atheta (Datomicra) whitehorsensis* Klimaszewski & Godin, **sp. n.**; 5) *Ocyusa yukonensis* Klimaszewski & Godin, **sp. n.**; 6) *Philhygra pseudolarsoni*Klimaszewski & Godin, **sp. n.**; 7) *Philhygra terrestris* Klimaszewski & Godin, **sp. n.**; 8) *Boreophilia davidgei* Klimaszewski & Godin, **sp. n.**; and 9) *Boreophilia herschelensis* Klimaszewski & Godin, **sp. n.**

## Introduction

Aleocharinae is the largest subfamily of Staphylinidae and embraces a wide variety of morphologically and ecologically diverse species that are poorly documented in Canada. This subfamily is widely distributed in North America and occurs in almost all terrestrial habitats. Most species are found in forests where they occur in leaf litter, under bark, in fungi, in moss and within the nests of ants, mammals and birds. In forest litter, the aleocharine fauna is a dominant group and part of a complex ecological web that is responsible for nutrient cycling, which ultimately contributes to forest productivity and resilience (Buse and Good 1993, Leschen 1993).

Currently, over 400 species of Aleocharinae in 92 genera are recorded from Canada and Alaska (Gouix and Klimaszewski 2007, Webster et al. 2009, Majka and Klimaszewski 2010, Klimaszewski et al. 2011). In a checklist of Canadian Coleoptera, Campbell and Davies (1991) recorded 59 species of Aleocharinae from the Yukon Territory. Gouix and Klimaszewski (2007) reported a fauna of 65 aleocharine species and in a more focused study of Yukon material, Klimaszewski et al. (2008) described 6 new species and provided 24 new territorial records, raising the total number of species to 95.

The present paper provides an updated review of aleocharine beetles from the Yukon Territory and constitutes important baseline data for monitoring the impact of invasive species, pollution, natural resource extraction and climate change. Additionally, the information and illustrations contained herein will make it possible to incorporate this diverse subfamily into ongoing Canadian biodiversity inventories including those in the Canadian Arctic.

## Materials and methods

Over 1,226 adults of Aleocharinae from the Yukon Territory were studied and most specimens were dissected to examine genitalia. The genital structures were dehydrated in absolute alcohol, mounted in Canada balsam on celluloid microslides and pinned with the specimens from which they originated. Photographs of the entire body and the genital structures were taken using an image processing system (Nikon SMZ 1500 stereoscopic microscope; Nikon Digit-like Camera DXM 1200F) and Adobe Photoshop software.

Morphological terminology mainly follows that used by Seevers (1978), Klimaszewski (1984) and Ashe (2001). The ventral part of the median lobe of the aedeagus is considered to be the part of the bulbus containing the foramen mediale, the entrance of the ductus ejaculatorius and the adjacent ventral part of the tubus of the median lobe with an internal sac and its structures (this part is referred to as the parameral side in some recent publications); the opposite side is referred to as dorsal. In the species descriptions, microsculpture refers to the surface of the upper forebody (head, pronotum and elytra).

Samples collected in this study include those from the Ecological Monitoring and Assessment Network (EMAN) plots. Two l ha plots, the Fireweed Drive (mixed pine and willow forest) and Cadet Camp (white spruce mature forest with feathermoss ground cover), have been reserved for long-term monitoring. All samples from these locations were collected from pitfall traps operating from late May to late September. Additional pitfall samples were collected by Donald Reid from early June to early August 2007, and early June to mid August 2008 at an alluvial fan on Hershel Island (dominated by *Carex* and grasses with some willows). All other sample collections were from organic litter sifting.

### Depository/institutional abbreviations:

CNCCanadian National Collection of Insects, Arachnids and Nematodes, Agriculture and Agri-Food Canada, Ottawa, Ontario, Canada


ECW>Environment Canada, Whitehorse, Yukon, Canada


LFCNatural Resources Canada, Canadian Forest Service, Laurentian Forestry Centre, René Martineau Insectarium, Québec City, Quebec, Canada


## Results

In this second recent survey of the Aleocharinae of the Yukon Territory, 125 species in 32 genera and 8 tribes are reported, including two tentative records. Nine species are newly described herein, 20 additional species constitute new territorial records and one species represents a new Canadian record. There are 6 adventive and 17 Holarctic species known from the territory and the status of two other species cannot yet be determined as belonging to either category. Adventive species constitute 4.8% of the total known aleocharine fauna of the Yukon.

## Discussion

The present survey increased the known Yukon aleocharine fauna from 95 to 125 species (Klimaszewski et al. 2008) and represents a significant contribution to the documentation of Canada’s entomofauna. Recent baseline surveys of Aleocharinae in other regions of Canada reported 203 species from the Maritime Provinces of Canada, of which 174 have been recorded in the past decade (Majka and Klimaszewski 2010), and 172 species from Newfoundland and Labrador (Klimaszewski et al. 2011).

Intensive sampling of the aleocharine fauna of the Yukon is continuing by the second author and undoubtedly many more species will be discovered in the future. The study of the Yukon fauna is particularly significant for understanding the shift in some species distributions in response to climate warming and for establishing baseline biodiversity data for northern Canada. Additionally, the occurrence of a species in the Yukon Territory otherwise known only from the eastern part of the country provides some evidence for a natural Holarctic distribution. Therefore, a survey of the biodiversity of the Yukon also contributes to our knowledge of species suspected of being adventive.

### Checklist of Aleocharinae species in the Yukon Territory

(* adventive species, ** Holarctic species, NTR=new territorial record for the Yukon Territory, NCR=new Canadian record; taxa in phylogenetic order).

**Order Coleoptera**

**Family Staphylinidae Latreille**

**Subfamily Aleocharinae Fleming**

I. Tribe Gymnusini Heer

***Gymnusa* Gravenhorst**

*Brevicollis* Group

1. *Gymnusa atra* Casey**

2. *Gymnusa konopackii* Klimaszewski

*Variegata* Group

3. *Gymnusa pseudovariegata* Klimaszewski

4. *Gymnusa smetanai* Klimaszewski**

5. *Gymnusa campbelli* Klimaszewski

II. Tribe Aleocharini Fleming

***Aleochara* Gravenhorst**

Subgenus *Aleochara* s. str.

6. *Aleochara* (s. str.) *assiniboin* Klimaszewski

7. *Aleochara* (s. str.) *lata* Gravenhorst*

8. *Aleochara* (s. str.) *sekanai* Klimaszewski

9. *Aleochara* (s. str.) *tahoensis* Casey

Subgenus *Coprochara*

10. *Aleochara (Coprochara) verna* Say

Subgenus *Xenochara*

11. *Aleochara (Xenochara) castaneipennis* Mannerheim

12. *Aleochara (Xenochara) fumata* Gravenhorst*

III. Tribe Oxypodini Thomson

***Calodera* Mannerheim**

13. *Calodera parviceps* (Casey) (NTR)

***Devia* Blackwelder**

14. *Devia prospera* (Erichson)**

***Gnathusa* Fenyes**

15. *Gnathusa caribou* Lohse

16. *Gnathusa eva*Fenyes (NTR)

17. *Gnathusa tenuicornis* Fenyes (NTR)

***Parocalea* Bernhauer**

18. *Parocalea nearctica* Lohse

19. *Parocalea pseudobaicalica* Lohse

***Neothetalia* Klimaszewski**

20. *Neothetalia canadiana* Klimaszewski

***Ocyusa* Kraatz**

21. *Ocyusa yukonensis* Klimaszewski & Godin, **sp. n.**

22. *Ocyusa canadensis* Lohse

***Oxypoda* Mannerheim**

*Convergens* Group

23. *Oxypoda pseudoconvergens* Klimaszewski & Godin

24. *Oxypoda canadensis* Klimaszewski (NTR)

*Lacustris* Group

25. *Oxypoda lacustris* Casey

26. *Oxypoda hiemalis* Casey

*Lucidula* Group

27. *Oxypoda lucidula* Casey

28. *Oxypoda demissa* Casey

*Operta* Group

29. *Oxypoda operta* Sjöberg* (NTR)

*Irrasa* Group

30. *Oxypoda irrasa* Mäklin

*Inimica* Group

31. *Oxypoda yukonensis* Klimaszewski & Godin

*Orbicollis* Group

32. *Oxypoda orbicollis* Casey

33. *Oxypoda frigida* Bernhauer

*Grandipennis* Group

34. *Oxypoda grandipennis* (Casey)

*Amica* Group

35. *Oxypoda amica* Casey (NTR)

***Phloeopora* Erichson**

36. *Phloeopora arctica* Lohse

***Brachyusa* Mulsant and Rey**

37. *Brachyusa helenae* (Casey) (NTR)

***Gnypeta* Thomson**

*Selmani* Group

38. *Gnypeta ashei* Klimaszewski

39. *Gnypeta brincki* Palm

40. *Gnypeta sellmani* Brundin**

*Caerulea* Group

41. Gnypeta caerulea** (C.R. Sahlberg)

IV. Tribe Hypocyphtini

***Cypha* Leach**

42. *Cypha inexpectata* Klimaszewski & Godin

V. Tribe Myllaenini Ganglbauer

***Myllaena* Erichson**

*Insomnis* Group

43. *Myllaena insomnis* Casey

VI. Tribe Homalotini Heer

***Gyrophaena* Mannerheim**

*Nana* Group

44. *Gyrophaena nana* (Paykull)**

45. *Gyrophaena neonana* Seevers

*Keeni* Group

46. *Gyrophaena keeni* Casey

*Pulchella* Group

47. *Gyrophaena criddlei* Casey (NTR) [tentative]

***Silusa* Erichson**

48. *Silusa californica* (Bernhauer)

VII. Tribe Placusini Mulsant and Rey

***Placusa* Erichson**

49. *Placusa tacomae* Casey

50. *Placusa vaga* Casey

VIII. Tribe Athetini Casey

***Acrotona* Thomson**

51. *Acrotona onthophila* Lohse

52. *Acrotona horwoodae* Klimaszewski & Godin, **sp. n.**

***Mocyta* Mulsant and Rey**

53. *Mocyta breviuscula* (Mäklin)

54. *Mocyta fungi* (Gravenhorst)*

***Strigota* Casey**

55. *Strigota ambigua* (Erichson) (NTR)

***Amischa* Thomson**

56. *Amischa praelonga* (Casey) (NCR, NTR)

57. *Amischa tersa* Casey [tentative]

***Atheta* Thomso*n***

Subgenus Atheta Thomson

58. *Atheta* (s. str.) *graminicola* (Gravenhorst)**

59. *Atheta* (s. str.) *martini* Lohse

Subgenus Pseudota Casey

*Klagesi* Group

60. *Atheta (Pseudota) klagesi* Bernhauer

Subgenus Oreostiba Ganglbauer

61. *Atheta (Oreostiba) sparreschneideri* Munster**

Subgenus Alaobia Thomson

62. *Atheta (Alaobia) ventricosa* Bernhauer

Subgenus Bessobia Thomson

63. *Atheta (Bessobia) cryptica* (Lohse)

Subgenus Dimetrota Mulsant and Rey

Altaica Group

64. *Atheta (Dimetrota) altaica* Bernhauer **

65. *Atheta (Dimetrota) nearctica* (Lohse)

Prudhoensis Group

66. *Atheta (Dimetrota) prudhoensis* (Lohse)

67. *Atheta (Dimetrota) burwelli* (Lohse)

68. *Atheta (Dimetrota) terranovae* Klimaszewski & Langor (NTR)

69. *Atheta (Dimetrota) caribou* (Lohse)

70. *Atheta (Dimetrota) strigosula* Casey

71. *Atheta (Dimetrota) pseudometlakatlana* Klimaszewski & Godin

Modesta Group

72. *Atheta (Dimetrota) pseudocrenuliventris* Klimaszewski

Campbelli Group

73. *Atheta (Dimetrota) smetanai* (Lohse)

74. *Atheta (Dimetrota) campbelli* (Lohse)

Fanatica Group

75. *Atheta (Dimetrota) fanatica* Casey(NTR)

76. *Atheta (Dimetrota) munsteri* Bernhauer**

Cadeti Group

77. *Atheta (Dimetrota) cadeti* Klimaszewski and Godin

Subgenus Rhagocneme Munster

78. *Atheta (Rhagocneme) subsinuata* (Erichson)*

Subgenus Datomicra Mulsant and Rey

79. *Atheta (Datomicra) dadopora* Thomson* or **

80. *Atheta (Datomicra) whitehorsensis* Klimaszewski & Godin, **sp. n.**

Subgenus Microdota Mulsant and Rey

81. *Atheta (Microdota) platonoffi* Brundin** (NTR)

82. *Atheta (Microdota) pratensis* (Mäklin) (NTR)

83. *Atheta (Microdota) microelytrata* Klimaszewski & Godin, **sp. n.**

84. *Atheta (Microdota) riparia* Klimaszewski & Godin, **sp. n.**

SUBGENUS UNCERTAIN

85. *Atheta brunswickensis* Klimaszewski

86. *Atheta capsularis* Klimaszewski

87. *Atheta remulsa* Casey

***Dinaraea* Thomson**

88. *Dinaraea angustula* (Gyllenhal)* (NTR)

89. *Dinaraea planaris* (Mäklin)

***Dochmonota* Thomson**

90. *Dochmonota rudiventris* (Eppelsheim)* or **

***Hydrosmecta* Thomso*n***

91. *Hydrosmecta pseudodiosica* Lohse

***Earota* Mulsant and Rey**

92. *Earota dentata* (Bernhauer)

***Emmelostiba* Pace**

93. *Emmelostiba microptera* (Lohse)

***Liogluta* Thomson**

94. *Liogluta aloconotoides* Lohse

95. *Liogluta granulosa* Lohse

96. *Liogluta trapezicollis* Lohse

97. *Liogluta nigropolita* (Bernhauer)

***Lypoglossa* Fenyes**

98. *Lypoglossa angularis* (Mäklin)

99. *Lypoglossa franclemonti* Hoebeke (NTR)

***Philhygra* Mulsant and Rey**

100. *Philhygra pseudopolaris* Klimaszewski and Langor [listed as *Philhygra polaris* (Bernhauer) by Lohse et al. 1990]

101. *Philhygra botanicarum* (Muona)**

102. *Philhygra pseudolarsoni*Klimaszewski & Godin, **sp. n.**

103. *Philhygra sinuipennis* Klimaszewski & Langor (NTR)

104. *Philhygra malleoides* Lohse

105. *Philhygra leechi* Lohse (NTR)

106. *Philhygra ripicoloides* Lohse

107. *Philhygra pseudoboreostiba* Lohse

108. *Philhygra juni* Lohse

109. *Philhygra clemens* (Casey) (NTR)

110. *Philhygra terrestris* Klimaszewski & Godin, **sp. n.**

111. *Philhygra jarmilae* Klimaszewski & Langor (NTR)

***Boreophilia* Benick**

112. *Boreophilia islandica* (Kraatz)**

113. *Boreophilia nearctica* Lohse

114. *Boreophilia blatchleyi* (Bernhauer & Scheerpeltz)

115. *Boreophilia venti* (Lohse)

116. *Boreophilia nomensis* (Casey) [Lohse et al. 1990 described this species as *Boreophilia caseyiana* Lohse, which was synonymized by Gusarov 2003]

117. *Boreophilia caseyi* Lohse

118. *Boreophilia insecuta* (Eppelsheim)**

119. *Boreophilia gelida* (J. Sahlberg)**

120. *Boreophilia herschelensis* Klimaszewski & Godin, **sp. n.**

121. *Boreophilia davidgei* Klimaszewski & Godin, **sp. n.**

***Boreostiba* Lohse**

122. *Boreostiba frigida* (J. Sahlberg)** [= *sibirica* sensu Lohse in Lohse et al. 1990]

123. *Boreostiba sibirica* (Mäklin)**

124. *Boreostiba parvipennis* (Bernhauer)

125. *Boreostiba lagunae* Lohse

### Systematic account of new records and new species of Aleocharinae from the Yukon territory

#### I. Tribe Oxypodini Thomson

##### Calodera parviceps

(Casey)

http://species-id.net/wiki/Calodera_parviceps

**Figures 1–6.**
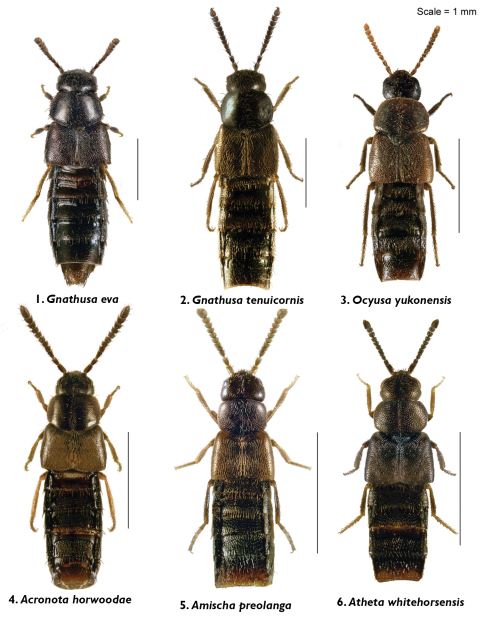
Body images in dorsal view: **1** *Gnathusa eva* Fenyes **2** *Gnathusa tenuicornis* Fenyes **3** *Ocyusa yukonensis* Klimaszewski & Godin, sp. n. **4** *Acrotona horwoodae* Klimaszewski & Godin, sp. n. **5** *Amischa praelonga* (Casey) **6** *Atheta (Datomicra) whitehorsensis* Klimaszewski & Godin, sp. n.

Figs 1–10 in Assing 2008

###### Distribution.

**Table:** 

Origin	Nearctic
Nearctic distribution	Canada: NS, NB, ON, YT; USA: RI
YT distribution	YUKON (NTR): Whitehorse, Paddy’s Pond, 60.7067, -135.0917, 6.V.2007, 649 m, litter sifting, mixed aspen and white spruce forest, B. Godin (ECW, LFC) 2 females
References	Casey 1894, Assing 2002, 2008

##### Gnathusa eva

Fenyes

http://species-id.net/wiki/Gnathusa_eva

Figs 1 13, 14

###### Distribution.

**Table:** 

Origin	Nearctic
Nearctic distribution	Canada (NTR): BC, YT; USA: CA
YT distribution	YUKON: Whitehorse, Granger subdivision, coniferous woodchip pile, 60.7097, -135.0996, 2.IX.2007, 661 m, pitfall trap, B. Godin (LFC) 1 male; same data except: 3.V.2008 (LFC, ECW) 4 males, 2 females
References	Fenyes 1910, 1920, Moore and Legner 1975, Majka and Klimaszewski 2008a

##### Gnathusa tenuicornis

Fenyes

http://species-id.net/wiki/Gnathusa_tenuicornis

Figs 2 15

###### Distribution.

**Table:** 

Origin	Nearctic
Nearctic distribution	Canada: YT, BC; USA: AK, CA
YT distribution	YUKON (NTR): Whitehorse, Paddy’s Pond, 60.7067, -135.0917, 6.V.2007, 649 m, litter sifting, mixed aspen and white spruce forest, B. Godin (ECW) 1 male
References	Fenyes 1921, Campbell and Davies 1991, Gouix and Klimaszewski 2007, Moore and Legner 1975, Klimaszewski and Winchester 2002

**Figures 7–12.**
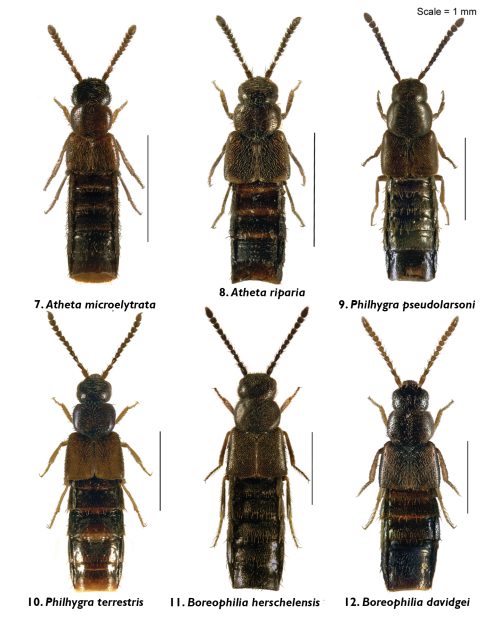
Body images in dorsal view: **7** *Atheta (Microdota) microelytrata* Klimaszewski & Godin, sp. n. **8** *Atheta (Microdota) riparia* Klimaszewski & Godin, sp. n. **9** *Philhygra pseudolarsoni* Klimaszewski & Godin, sp. n. **10** *Philhygra terrestris* Klimaszewski & Godin, sp. n. **11** *Boreophilia herschelensis* Klimaszewski and Godin, sp. n. **12** *Boreophilia davidgei* Klimaszewski & Godin, sp. n.

##### Ocyusa yukonensis

Klimaszewski & Godin sp. n.

urn:lsid:zoobank.org:act:CAF7FE71-43FD-4C09-9B9C-FE58D3D72F29

http://species-id.net/wiki/Ocyusa_yukonensis

Figs 3 16 32, 33

###### Holotype

(male)**.** Canada, Yukon, EMAN Plot (Ecological Monitoring and Assessment Network), mature white spruce and feathermoss forest, 60.5963, -134.9522, 8.VII.2003, 738 m, yellow pitfall trap (LMKM31Y), (LFC).

###### Paratype.

Yukon, EMAN Plot, 60.5963, -134.9522, 24.VII.2003, 738 m, black pitfall trap (LMKM31B), (ECW) 1 male.

###### Etymology.

*Yukonensis -* a Latin adjective derived from the Yukon Territory, Canada.

###### Diagnosis.

Body small, subparallel, robust, uniformly dark brown, almost black; length 2.8–3.0 mm; head round in outline and almost as wide as pronotum; antennae with article 4 subquadrate, 5–10 moderately transverse, increasingly wider apicad; pronotum transverse, angular posteriad and slightly narrower than maximum width of elytra; abdomen subparallel, at base as wide as elytra (Fig. 3). MALE: male tergite 8 widely truncate apically (Fig. 32); sternite 8 slightly produced at apex (Fig. 33); median lobe of aedeagus as illustrated (Fig. 16). FEMALE: unknown.

###### Distribution.

This native Nearctic species is known only from the type locality in the Yukon.

###### Bionomics.

Two adults were collected in July.

**Figures 13–21.**
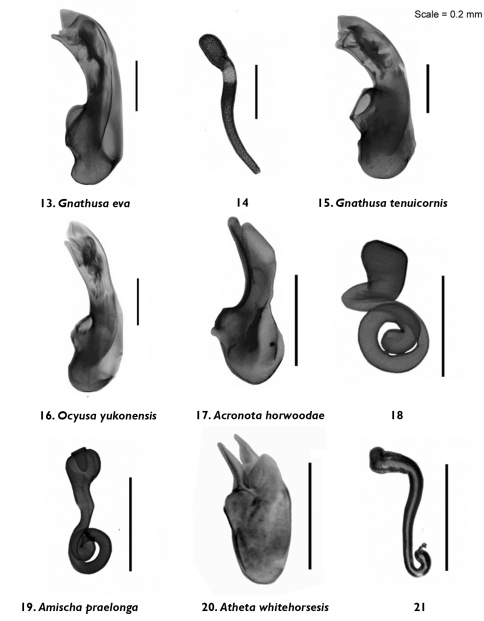
Median lobe of aedeagus and spermatheca in lateral view of *Gnathusa eva* Fenyes **13, 14** *Gnathusa tenuicornis* Fenyes **15** *Ocyusa yukonensis* Klimaszewski & Godin, sp. n. **16** *Acrotona horwoodae* Klimaszewski & Godin, sp. n. **17, 18** *Amischa praelonga* (Casey) **19** *Atheta (Datomicra) whitehorsensis* Klimaszewski & Godin, sp. n. **20, 21**.

##### Oxypoda canadensis

Klimaszewski

http://species-id.net/wiki/Oxypoda_canadensis

Figs 5, 41, 80–82, 171, 203, 204, 209, 210, in Klimaszewski et al. 2006

###### Distribution.

**Table:** 

Origin	Nearctic
Nearctic distribution	Canada: NL, QC, ON, MB, AB, YT, NT; USA: AK, NH
YT distribution	YUKON (NTR): Whitehorse, Paddy’s Pond, 60.7067, -135.0917, 6.V.2007, 649 m, litter sifting, mixed aspen and white spruce forest, B. Godin (ECW) 1 male, 1 female; Watson Lake - Watson Creek, 60.1272, -128.805, 7.VII.2008, 697 m, deciduous debris soil sifting, B. Godin (ECW) 1 male, 2 females; Contact Creek, 65 km E Watson Lake; 59.9995, -127.7241,8.VI.2008, 621 m, litter sifting, creek bank, B. Godin (ECW) 1 male; Upper Liard, Albert Creek, 60.0522, -128.928, 8.VII.2008, 619 m, deciduous forest soil sifting, B. Godin (ECW, LFC) 3 males, 4 females
References	Klimaszewski et al. 2006, Gouix and Klimaszewski 2007, Klimaszewski et al. 2011

##### Oxypoda operta

Sjöberg* or **

http://species-id.net/wiki/Oxypoda_operta

Figs 16, 52, 104, 105, 181, 245, 246, 249, 250, in Klimaszewski et al. 2006

###### Distribution.

**Table:** 

Origin	Holarctic or Palaearctic
Nearctic distribution	Canada: NL, NS, QC, ON, AB, YT; USA: NH
YT distribution	YUKON (NTR): Watson Lake - Watson Creek, 60.1272, -128.805, 4.VI.2008, 697 m, deciduous debris, soil sifting, B. Godin (ECW) 1 male, 1 female
References	Smetana 2004, Klimaszewski et al. 2006, Gouix and Klimaszewski 2007, Majka and Klimaszewski 2010, Klimaszewski et al. 2011

##### Brachyusa helenae

(Casey)

http://species-id.net/wiki/Brachyusa_helenae

Figs 48, 49, 222a-c, in Klimaszewski et al. 2011

###### Distribution.

**Table:** 

Origin	Nearctic
Distribution	**Canada:** NL, YT, NT; **USA:** AK, MT
YT distribution	YUKON **(NTR):** Nisutlin Wildlife Area, 60.2317, -132.5632, 17.IX.2007, 679 m, pitfall – Willow stand #2 (ECW, LFC) 2 females
References	Casey 1911, Campbell and Davies 1991, Gouix and Klimaszewski 2007, Klimaszewski et al. 2011

#### II. Tribe Homalotini Heer

##### Gyrophaena criddlei

Casey

http://species-id.net/wiki/Gyrophaena_criddlei

Figs 16, 107–110, in Klimaszewski et al. 2009

###### Distribution.

**Table:** 

Origin	Nearctic
Distribution	Canada: NL, NB, MB, YT
YT distribution	YUKON (NTR): Watson Lake – Watson Creek, 60.12723, -128.8053, 16. VIII.2007, 697 m, mushrooms, B. Godin (LFC) 1 female; Granger, 60.7078, 135.0971, 25.VIII.2007, 657 m, B. Godin (LFC) 1 female.
References	Seevers 1951, Gouix and Klimaszewski 2007, Klimaszewski et al. 2009, Klimaszewski et al. 2011

###### Comments.

The two females are tentatively identified as *Gyrophaena criddlei* but a male is needed for positive confirmation of this species in the Yukon Territory.

#### III. Tribe Athetini Casey

##### Acrotona horwoodae

Klimaszewski & Godin sp. n.

urn:lsid:zoobank.org:act:D5CA8598-36E8-40B4-AEAD-20D013A6964E

http://species-id.net/wiki/Acrotona_horwoodae

Figs 4 17, 18 34–37

###### Holotype

**(male).** Canada, Yukon, Whitehorse, Paddy’s Pond, 60.7067, -135.0917, 27.V.2008, 649 m, litter sifting, mixed aspen and white spruce forest, B. Godin (LFC).

###### Paratype

(female)**.** Same data as the holotype (ECW).

###### Etymology.

This species name is dedicated to Denise Horwood, wife of the second author, who assisted him in numerous aleocharine sample collections.

###### Diagnosis.

Body narrowly oval, moderately convex, uniformly black, punctation on forebody fine, dense and not asperate, microsculpture fine but not pronounced; length 2.4 mm; head narrower than pronotum, ratio of maximum width of head to maximum width of pronotum 0.7; antennal articles 7–10 slightly transverse; pronotum moderately transverse, ratio of maximum width to length 1.4, about as wide as elytra; elytra at suture about as long as pronotum; abdomen slightly narrowed posteriad (Fig. 4). MALE: tergite 8 moderately elongate and truncate apically (Fig. 34); sternite 8 widely arcuate apically (Fig. 35); median lobe of aedeagus as illustrated (Fig. 17). FEMALE: tergite 8 moderately elongate and truncate apically, base not sinuate (Fig. 36); sternite 8 widely arcuate apically, base not sinuate (Fig. 37); spermatheca with capsule tulip-shaped and stem coiled posteriorly (Fig. 18).

**Bionomics.** The specimens were found by sifting forest litter in May.

**Comments.** The shape of the median lobe of the aedeagus and the spermatheca of *Acrotona horwoodae* are different from all recorded species of Nearctic *Acrotona*, and they are generally similar to those of the Palaearctic species *Acrotona aterrima* Gravenhorst, which is brown and has a much broader body.

##### Strigota ambigua

(Erichson)

http://species-id.net/wiki/Strigota_ambigua

Figs 88, 261a-c, in Klimaszewski et al. 2011

###### Distribution.

**Table:** 

Origin	Nearctic
Distribution	Canada: NL, NS, PE, YT; USA: CA, CO, CT, IA, KS, MA, MO, NC, NJ, NV, NY, TX
YT distribution	YUKON (NTR): Whitehorse, 60.7328, -135.0986 18.VI.2007, 717 m, hand collected, parking lot asphalt, (ECW) 1 female
References	Bernhauer 1907, Gusarov 2003, Gouix and Klimaszewski 2007, Majka et al. 2008b, Majka and Klimaszewski 2010, Klimaszewski et al. 2011

##### Amischa praelonga

(Casey)

http://species-id.net/wiki/Amischa_praelonga

Figs 5 19 38, 39

###### Distribution.

**Table:** 

Origin	Nearctic
Distribution	Canada (NTR): YT; USA: WY
YT distribution	YUKON (NTR): Whitehorse, McIntyre Creek, 60.7398, -135.1462, 25.IV.2007, 744 m, litter sifting, willow stand by creek bank, B. Godin (ECW, LFC) 2 females; EP Impact, south, 60.7336, -135.0946,19.VII.2001, 695 m, pitfall trap, disturbed land, grasses, B. Godin (ECW, LFC) 3 females
References	Casey 1894

###### Comments.

Two additional *Amischa* morphotypes were recognized in the Yukon material on the basis of external body characters and the shape of the spermatheca. They are not included in this account because they are difficult to associate with any of the recorded species. The first morphospecies is represented by three narrowly elongate bicoloured specimens with the head and 4–5 basal abdominal tergites almost black, with the pronotum brown and the appendages and posterior of the elytra light brown, and with the spermathecal capsule moderately elongate with a moderately long apical invagination. The second morphospecies is represented by three specimens, which are broader, with the body uniformly dark brown to almost black, and the spermathecal capsule broader and shorter apically and with a longer apical invagination. Both groups have the apex of tergite 8 deeply notched. We need more specimens and representatives of both sexes to establish the status of these morphotypes.

##### Atheta (Dimetrota) terranovae

Klimaszewski & Langor

http://species-id.net/wiki/Atheta_terranovae

Figs 107, 280a–c, 407a–d, in Klimaszewski et al. 2011

###### Distribution.

**Table:** 

Origin	Nearctic
Distribution	Canada: NL, YT
YT distribution	YUKON (NTR): Whitehorse, Granger, 60.7078, -135.0971, 1.VIII.2007, 657 m, mushrooms, B. Godin (ECW) 2 females; same data except: 60.7366, 135.097, 15.VIII.2008, 743 m, pitfall trap, ski trail, birch stand, B.Godin (ECW) 1 male; EMAN Plot, Fireweed Dr., 60.6014,-134.9387, 8.VIII.2006, 772 m, pitfall trap, mixed pine and willow forest (ECW) 1 male; same data except: 23.VII.2006 (ECW) 1 female; EMAN Plot, Cadet Camp, 60.5951, -134.9499, 23.VIII.2006, 760 m, pitfall trap, mature white spruce and feathermoss forest, (ECW) 1 female
References	Klimaszewski et al. 2011

##### Atheta (Dimetrota) fanatica

Casey

http://species-id.net/wiki/Atheta_fanatica

Figs 134, 307a–c, in Klimaszewski et al. 2011

###### Distribution.

**Table:** 

Origin	Nearctic
Distribution	Canada: NL, NS, NB, QC, YT, BC; USA: AK, NV
YT distribution	YUKON (NTR): Whitehorse, Paddy’s Pond, 60.7067, -135.0917, 20.V.2007, 649 m, litter sifting, B. Godin (ECW) 1 male; Whitehorse, Granger, 60.7078, -135.0971, 5.VIII.2007, 657 m, soil sifting, B. Godin (ECW) 1 male; same data except: 27.IX.2008, compost (LFC) 1 male, 1 female
References	Campbell and Davies 1991, Casey 1910, 1911, Moore and Legner 1975, Majka et al. 2006 [as irrita], Webster et al. 2009 [as irrita], Majka and Klimaszewski 2010 [as irrita], Klimaszewski et al. 2011

##### Atheta (Dimetrota) whitehorsensis

Klimaszewski & Godin sp. n.

urn:lsid:zoobank.org:act:9ACD0F86-341A-4855-925A-51104BB8C8F4

http://species-id.net/wiki/Atheta_whitehorsensis

Figs 6 20, 21 40–43

###### Holotype (male).

Canada, Yukon, Whitehorse, Granger, 60.7078, -135.0971, 25.VIII.2007, 657 m, soil sifting, black spruce stand, AWT, B. Godin (LFC).

###### Paratype.

Canada, Yukon, Whitehorse, Granger, 60.7078, -135.0971, 5.VIII.2007, 657 m, soil sifting, black spruce stand, AWT, B. Godin (ECW) 1 female.

###### Etymology.

The specific name derives from the name of the type locality, which is Whitehorse, Yukon.

###### Diagnosis.

Body narrowly oval, dark brown to black, with bases of antennae and legs rust-brown, surface matte, with asperate dense punctation on forebody and strong meshed microsculpture (Fig. 6); length 1.9–2.0 mm; head narrower than pronotum and elytra, with short postocular area, eyes large and slightly protruding; antennae slender, slightly incrassate apically, article 4 subquadrate, 5 slightly elongate and 6–10 slightly to strongly transverse; pronotum strongly transverse and broadest in the middle; elytra transverse, longer than pronotum; abdomen broadly arcuate laterally (Fig. 6). MALE: tergite 8 transverse and truncate apically (Fig. 40); sternite 8 widely rounded apically (Fig. 41); median lobe of aedeagus with venter of tubus straight and short, and apex sharply produced (Fig. 20). FEMALE: tergite and sternite 8 truncate apically (Figs 42, 43); spermatheca with pipe-shaped capsule and long stem hooked posteriorly (Fig. 21).

This species is similar externally to *Atheta (Dimetrota) hampshirensis* Bernhauer and *Atheta (Datomicra) dadopora* Thomson but differs in the shape of the spermatheca and median lobe of the aedeagus, and has a broader body than the latter species.

###### Distribution.

This native Nearctic species is known only from the type locality in the Yukon Territory.

###### Bionomics. 

Adults were captured by sifting soil in a black spruce stand.

##### Atheta (Microdota) platonoffi

Brundin**

http://species-id.net/wiki/Atheta_platonoffi

Figs 127, 300a-c, 423, in Klimaszewski et al. 2011

###### Distribution.

**Table:** 

Origin	Holarctic
Distribution	Canada: NL, NS, NB, ON, AB, BC, YT; USA: AK
YT distribution	YUKON (NTR): Whitehorse, Granger, 60.7078, -135.0971, 25.VIII.2007, 657 m, soil sifting, black spruce stand, B. Godin (ECW, LFC) 3 males, 2 females; same data except: 1.VIII.2008, mushrooms (ECW, LFC) 3 males; 16.VIII.2007, mushrooms (ECW) 1 female; Upper Liard, Albert Creek, 60.0522, -128.928, 8.VII.2007, 699 m, deciduous debris, soil sifting, B. Godin (ECW) 1 female
References	Klimaszewski et al. 2005, Gouix and Klimaszewski 2007, Majka et al. 2008b, 2010, Klimaszewski et al. 2011

##### Atheta (Microdota) pratensis

(Mäklin)

http://species-id.net/wiki/Atheta_pratensis

Figs 128, 301a–c, 428, in Klimaszewski et al. 2011

###### Distribution.

**Table:** 

Origin	Nearctic
Distribution	Canada: NL, YT; USA: AK
YT distribution	YUKON (NTR): Tagish, Tagish Lake; 60.2658, -134.2873, 20.VIII.2007, 654 m, mushroom, B. Godin (ECW) 1 male
References	Mäklin 1853, Klimaszewski et al. 2011

##### Atheta (Microdota) microelytrata

Klimaszewski & Godin sp. n.

urn:lsid:zoobank.org:act:A75DCD78-E696-4AE7-8E8C-ACAF8F3B3F7E

http://species-id.net/wiki/Atheta_microelytrata

Figs 7 22, 23 44–47

###### Holotype

(male)**.** Canada, Yukon, Whitehorse, Takhini, hotsprings, 60.8769, -135.3596, 30.IV.2009, 716 m, aspen litter – soil sifting, B. Godin (LFC).

###### Paratypes

**.** Canada, Yukon, Whitehorse, Takhini, hotsprings, 60.8769, -135.3596, 19.IX.2009, 716 m, alder/willow litter, soil sifting, B. Godin (ECW) 2 males; same data except: 3.V.2009 (ECW, LFC) 2 females.

###### Etymology.

The specific name derives from the word micro, meaning small, and elytra,in allusion to the small and short elytra of this species.

###### Diagnosis.

**Figures 22–31.**
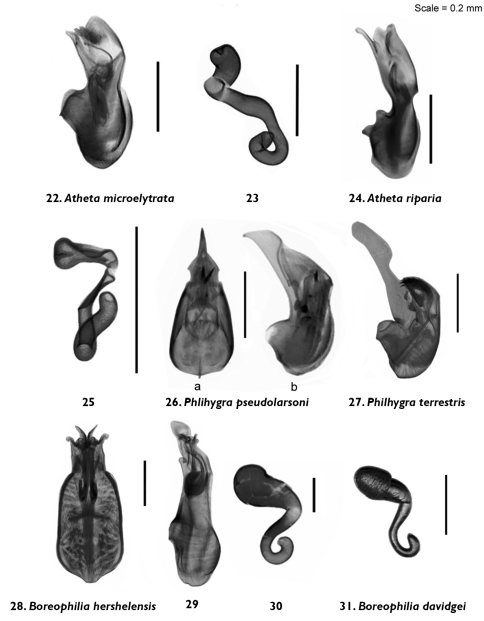
Median lobe of aedeagus and spermatheca (view as specified) of *Atheta (Microdota) microelytrata* Klimaszewski and Godin, sp. n. **22** lateral **23** lateral; *Atheta (Microdota) riparia* Klimaszewski & Godin, sp. n. **24** lateral **25** lateral; *Philhygra pseudolarsoni* Klimaszewski & Godin, sp. n. **26** lateral; *Philhygra terrestris* Klimaszewski & Godin, sp. n. **27** lateral; *Boreophilia herschelensis* Klimaszewski & Godin, sp. n. **28** dorsal **29** lateral **30** lateral; *Boreophilia davidgei* Klimaszewski & Godin, sp. n. **31** lateral.

Body narrowly subparallel; dark brown, with bases of antennae and legs rust-brown; strongly glossy, with fine and moderately dense punctation on forebody and strong, meshed microsculpture (Fig. 7); head as wide as pronotum and elytra, with long postocular area, eyes moderately small and slightly protruding; antennae slender, slightly incrassate apicad, articles 4–5 subquadrate and 6–10 slightly to strongly transverse; pronotum narrower at base and broadening apicad; elytra transverse, shorter than pronotum; abdomen widest subapically; length 1.9–2.0 mm (Fig. 7). MALE: tergite 8 truncate apically and with crenulation scarcely visible (Fig. 44); sternite 8 widely rounded apically (Fig. 45); median lobe of aedeagus with apex narrow and ventrally produced, athetine bridge well developed (Fig. 22). FEMALE: tergite 8 truncate apically (Fig. 46); sternite 8 truncate and slightly emarginate medially (Fig. 47); spermatheca with pipe-shaped capsule and long, posteriorly-coiled stem (Fig. 23).

This species bears some superficial external similarity to *Geostiba* and *Emmelostiba* but has typical *Atheta*-like genitalia.

###### Distribution.

This native Nearctic species is known only from the type locality in the Yukon Territory.

###### Bionomics.

Adults were found in aspen, alder and willow litter in March, May and September.

##### Atheta (Microdota) riparia

Klimaszewski & Godin sp. n.

urn:lsid:zoobank.org:act:BC82DFB4-F60B-4758-9860-BC23B2F3D6DC

http://species-id.net/wiki/Atheta_riparia

Figs 8 24, 25 48–51

###### Holotype

(male)**.** Canada, Yukon, Whitehorse, Paddy’s Pond, 60.7067, -135.0917, 16.IX.2007, 649 m, litter sifting, mixed aspen and white spruce forest, B. Godin (LFC).

###### Paratype.

Same data as the holotype (ECW) 1 male.

###### Non-type.

Canada, Yukon, Watson Lake, Watson Creek, 60.12723, -128.8053, 16.VIII.2007, 697 m, mushrooms, B. Godin (LFC) 1 female.

###### Etymology.

The name of this species derives from the Latin adjective *riparius, -a, -um*,in allusion to the wet litter where the types were found.

###### Diagnosis.

Body small and narrow, subparallel; black, with tarsi reddish-brown; moderately glossy, with fine, dense punctation and meshed microsculpture on forebody (Fig. 8); head approximately as wide as pronotum, depressed medially, eyes slightly protruding; antennae slender, slightly incrassate apicad, articles 4–10 slightly to strongly transverse; pronotum emarginate laterally; elytra broader and longer at suture than pronotum; head, pronotum and base of abdomen of the same width; sides of abdomen subparallel; length 1.9–2.0 mm (Fig. 8). MALE: tergite 8 truncate apically and with smooth margin (Fig. 48); sternite 8 widely rounded apically (Fig. 49); median lobe of aedeagus with apex narrow and ventrally produced (Fig. 24). FEMALE (non-paratype): tergite 8 truncate apically (Fig. 50); sternite 8 broadly rounded apically (Fig. 51); spermatheca slightly distorted but with club-shaped capsule and posteriorly-twisted stem (Fig. 25).

This species differs from other Nearctic *Microdota* by the combination of body shape, strongly punctate surface and the shape of the median lobe of the aedeagus and spermatheca.

**Distribution.** This native Nearctic species is known only from the Yukon Territory but it is probably more widely distributed in northern Canada.

**Bionomics.** The two males were captured in September in wet, organic litter and the female was found in mushrooms in mid-August.

##### Dinaraea angustula

(Gyllenhal)*

http://species-id.net/wiki/Dinaraea_angustula

Figs 141, 314a–c, 442, in Klimaszewski et al. 2011

###### Distribution.

**Table:** 

Origin	Palaearctic
Distribution	Canada: NL, NS, NB, PE, QC, ON, AB, YT; USA: CA, NY
YT distribution	YUKON (NTR): EMAN plot, Cadet Camp, 60.5951, -134.9499, 26.V.2006, 760 m, pitfall trap, mature white spruce and feathermoss forest, B. Godin (LFC) 1 male
References	Moore and Legner 1975, Muona 1984, Smetana 2004, Klimaszewski et al. 2007, Gouix and Klimaszewski 2007, Webster et al. 2009, Majka et al. 2008b, 2010, Klimaszewski et al. 2011

##### Lypoglossa franclemonti

Hoebeke

http://species-id.net/wiki/Lypoglossa_franclemonti

Figs 154, 328a–c, 455, in Klimaszewski et al. 2011

###### Distribution.

**Table:** 

Origin	Nearctic
Distribution	Canada: NL, NB, NS, QC, ON, MB, AB, YT, NT; USA: ME, NH, NY, VT
YT distribution	YUKON (NTR): Upper Liard, Albert Creek, 60.0522, -128.9279, 3.VI.2007, 699 m, deciduous litter sifting, B. Godin (ECW, LFC) 4 males, 2 females; same data except: 4.VI.2007 (ECW, LFC) 1 male, 2 females, 7.VII.2008 (ECW, LFC) 2 males; Watson Lake, Watson Creek, 60.12723, -128.8053, 16.VIII.2007, 697 m (ECW) 1 male
References	Hoebeke 1992, Gusarov 2004, Gouix and Klimaszewski 2007, Klimaszewski et al. 2011

##### Philhygra pseudolarsoni

Klimaszewski & Godin sp. n.

urn:lsid:zoobank.org:act:64A996FC-47AE-453A-A112-B57D0C0D950F

http://species-id.net/wiki/Philhygra_pseudolarsoni

Figs 9 26 52–55

###### Holotype (male).

Canada, Yukon, Whitehorse, Paddy’s Pond, 60.7067, -135.0917, 26.V.2007, 649 m, litter sifting, mixed aspen and white spruce forest, B. Godin (LFC).

###### Paratypes.

same label data as the holotype (ECW) 1 male; Watson Lake, Watson Creek, 60.1272, -128.8053, 4.VI.2007,697 m, deciduous forest soil sifting, B. Godin (ECW) 1 male, 1 female.

###### Etymology.

This species name derives from the specific name *larsoni* (*Philhygra larsoni* Klimaszewski and Langor), and the prefix *pseudo* (false) in relation to the similarity of the two species in external and, to a lesser degree, genitalic morphology.

###### Diagnosis.

Body narrowly subparallel, uniformly black or black with legs and sutural part of elytra reddish-brown (Fig. 9); moderately glossy, with fine, dense punctation and meshed microsculpture on forebody; head round, distinctly narrower than pronotum, with eyes as long as postocular region of head; antennae slender with articles 4–5 elongate, 6–10 subquadrate to slightly transverse; pronotum slightly transverse and almost as wide as elytra; elytra at suture as long as or slightly longer than pronotum; length 2.9–3.0 mm (Fig. 9). MALE: tergite 8 widely arcuate apically (Fig. 52); sternite 8 elongate and rounded apically (Fig. 53); median lobe of aedeagus with apex triangularly produced in lateral view (Fig. 26).

Female. tergite 8 truncate apically (Fig. 54); sternite 8 produced medially (Fig. 55); pygidium with ventral structure weakly sclerotized.

###### Distribution.

This species is known only from Whitehorse and Watson Lake in the Yukon Territory.

###### Bionomics

**.** This species was collected in May and June from ground litter.

###### Comments.

*Philhygra pseudolarsoni* is similar in both external morphology and genitalia to *Philhygra larsoni* Klimaszewski and Langor. However, it may be distinguished from *Philhygra larsoni* by the smaller and darker body, quadrate or transverse antennal articles 4–10 and by the median lobe of the aedeagus with a more elongate apical part of the tubus in lateral view.

##### Philhygra sinuipennis

Klimaszewski & Langor

http://species-id.net/wiki/Philhygra_sinuipennis

Figs 161, 335a, b, 462a, b, in Klimaszewski et al. 2011

###### Distribution.

**Table:** 

Origin	Nearctic
Distribution	Canada: NL, YT
YT distribution	YUKON (NTR): Watson Lake, Watson Creek, 60.1272, -128.8053, 4.VI.2007, 697 m, deciduous litter sifting, B. Godin (ECW, LFC) 2 males
References	Klimaszewski et al. 2011

##### Philhygra leechi

Lohse

http://species-id.net/wiki/Philhygra_leechi

Figs 118, 119, in Lohse et al. 1990

###### Distribution.

**Table:** 

Origin	Nearctic
Distribution	Canada: MB, YT, NT
YT distribution	YUKON (NTR): Nisutlin Wildlife Area, 60.2317, -132.5632, 21.VIII.2007, 679 m, pitfall – Willow stand # 2, B. Godin (LFC) 1 male.
References	Lohse et al. 1990, Gouix and Klimaszewski 2007

##### Philhygra terrestris

Klimaszewski & Godin sp. n.

urn:lsid:zoobank.org:act:246EBFF8-C0AE-43D6-98D9-C99289EE7B47

http://species-id.net/wiki/Philhygra_terrestris

Figs 10 27 56, 57

###### Holotype

(male)**.** Canada, Yukon, Whitehorse, Paddy’s Pond, 60.7067, -135.0917, 26.V.2007, 649 m, litter sifting, mixed forest (aspen and white spruce), B. Godin (LFC).

**Etymology.** This species name is an adjective that derives from the Latin word *terra* (ground, earth, soil).

**Diagnosis.** Body narrowly subparallel, head and abdomen black, pronotum and elytra brown, basal article of antenna and legs yellowish (Fig. 10); strongly glossy, with fine, dense punctation and meshed microsculpture on forebody; head round, distinctly narrower than pronotum with eyes as long as postocular region of head; antennae slender with articles 4–5 elongate, 6–10 subquadrate; pronotum slightly transverse and almost as wide as elytra; elytra at suture slightly longer than pronotum; length 2.9–3.0 mm (Fig. 10). MALE: tergite 8 widely arcuate apically (Fig. 56); sternite 8 elongate and rounded apically (Fig. 57); aedeagus with apex of median lobe broadly produced and with tubus constricted basally in lateral view (Fig. 27).

Female. unknown.

**Distribution.** This species is known only from Whitehorse in the Yukon but it may be more widely distributed in the boreal zone of Canada and Alaska.

**Bionomics.** This species was collected in May from ground litter.

**Comments.** This species is unique in the shape of the median lobe of the aedeagus in lateral view.

##### Philhygra jarmilae

Klimaszewski & Langor

http://species-id.net/wiki/Philhygra_jarmilae

Figs 159, 333a, b, 460a-d, in Klimaszewski et al. 2011

###### Distribution.

**Table:** 

Origin	Nearctic
Distribution	Canada: YT, NL
YT distribution	YUKON (NTR): Albert Creek, 60.0522, -128.9279, 3.VI.2007, soil sifting, willow stand, B. Godin (LFC) 1 male.
References	Gouix and Klimaszewski 2007, Klimaszewski et al. 2011

##### Boreophilia herschelensis

Klimaszewski & Godin sp. n.

urn:lsid:zoobank.org:act:DD1259D2-69BE-4A73-B26F-DEA59F7F47D0

http://species-id.net/wiki/Boreophilia_herschelensis

Figs 11 28–30 58–61

###### Holotype

(female)**.** Canada, Yukon, Herschel Island, 69.5706, -138.902, 13.VI.2007, 5 m, pitfall trap, site dominated by *Carex* and grasses with presence of willows (ATOR) – alluvial fan, D.G. Reid (LFC).

###### Paratypes. 

Labeled as the holotype except: 1–3.VI.2007 (ECW) 1 male; 7.VI.2007 (ECW) 2 males; 10.VI.2007 (CNC) 1 male; 15.VI.2007 (ECW) 1 female; 17.VI.2007 (ECW) 1 male, 1 female; 19.VI.2007 (ECW) 1 female; 16.VII.2007 (LFC) 1 male, 1 female; 21.VII.2007 (ECW) 2 females; 31.VII.2007 (LFC) 1 male; 7.VI.2008 (ECW) 2 females; 7.VII.2008 (ECW) 2 females; 15.VII.2008 (ECW) 1 female; 11.VIII.2008 (ECW) 1 female.

###### Etymology.

Named for the type locality, Herschel Island.

###### Diagnosis.

Body narrow, subparallel, head and pronotum about the same width, elytra and abdomen slightly wider, uniformly black (Fig. 11); surface matte except for slightly glossy abdomen; pubescence fine, punctation weak and moderately dense, meshed microsculpture pronounced on forebody; head round, slightly flattened medially and with eyes about as long as postocular region of head; antennae slender, articles 4–5 slightly elongate, 6–10 subquadrate, last article elongate; pronotum transverse, narrower at base and widest at middle; elytra at suture slightly longer than or as long as pronotum; abdomen subparallel for most of its length; length 2.8–3.0 mm (Fig. 11). MALE: tergite 8 transverse and truncate apically (Fig. 58); sternite 8 slightly elongate and rounded apically (Fig. 59); median lobe of aedeagus with straight tubus in lateral view and with apex short and narrow (Fig. 29), dorsal aspect as illustrated (Fig. 28). FEMALE: tergite 8 transverse and truncate apically (Fig. 60); sternite 8 slightly elongate and rounded apically (Fig. 61); spermatheca S-shaped, capsule consisting of a globular apical part with a small invagination, stem sinuate (Fig. 30).

The following combination of characters distinguishes this species from other congeners: narrow, subparallel and uniformly black body, integument of forebody matte and with dense microsculpture, median lobe of aedeagus narrow apically and spermatheca S-shaped.

###### Distribution.

This Nearctic species is known only from the type locality on Herschel Island, Yukon.

###### Bionomics.

Adults were collected in June and July on an alluvial fan.

###### Comments.

This species is superficially similar to *Boreophilia nomensis* Casey (=*Boreophilia caseyiana* Lohse) but differs by its uniformly black body and aedeagus with evenly narrow apical part of median lobe in lateral view.

##### Boreophilia davidgei

Klimaszewski & Godin sp. n.

urn:lsid:zoobank.org:act:6561B1F8-3DFD-4745-B5F3-7A3131152979

http://species-id.net/wiki/Boreophilia_davidgei

Figs 12 31 62, 63

###### Holotype

(female). Canada, Yukon, EMAN Plot, Cadet Camp, 60.5951, -134.9499, 20.IX.2006, 760 m, pitfall trap, mature white spruce and feathermoss forest, coll. EP Yukon, AJK (LFC).

###### Paratypes.

Canada, Yukon, EMAN Plot, Cadet Camp, 60.5951, -134.9499, 29.V.2006, 760 m, pitfall trap, mature white spruce and feathermoss forest, EP Yukon, AHW (ECW) 1 female; same data except: 15.V.2002, JF (ECW) 1 female; 12.VI.2002, EV (ECW) 1 female; 18.X.2002, FD (CNC) 2 females; 8.VII.2003, LMK31Y. LJ (ECW) 1 female; Fireweed Dr., 60.6014, -134.9387, 23.IX.2000, 772 m, pitfall trap, mixed pine and willow forest, EP Yukon (ECW) 1 female; Whitehorse, Granger, 60.7078, -135.0971, 5.VIII.2007, 657 m, soil sifting, black spruce stand, B. Godin (ECW, LFC) 2 females; same data except: 25.VIII.2007 (LFC) 1 female; Whitehorse, Paddy’s Pond, 60.7067, -135.0917, 16.IX.2007, 649 m, litter sifting, mixed aspen and white spruce forest, B. Godin (ECW) 1 female; Upper Liard, Albert Creek, 60.0522, -128.928, 8.VII.2000, 699 m, deciduous litter sifting, B. Godin (ECW, LFC) 2 females.

###### Etymology.

Named for Douglas Davidge, biological technician (ECW), who supported the second author in his work for 20 years.

###### Diagnosis.

Body narrow, subparallel, head narrower than pronotum, elytra and abdomen slightly wider, uniformly brown with appendages yellowish-brown and antennae yellow, or with head and abdomen dark brown and rest of body light brown (Fig. 12); surface moderately glossy; pubescence fine, punctation weak and moderately dense, meshed microsculpture pronounced on forebody; head round, slightly flattened medially and with eyes about as long as postocular region of head; antennae slender, articles 4–5 slightly elongate, 6–10 subquadrate to slightly transverse, last article elongate; pronotum transverse, widest in basal half; elytra at suture slightly longer than pronotum; abdomen broadly arcuate laterally; length 2.8–2.9 mm (Fig. 12). MALE: unknown. FEMALE: tergite 8 transverse and truncate apically (Fig. 62); sternite 8 slightly elongate and rounded apically (Fig. 63); spermatheca S-shaped, capsule elongate, stem short and sinuate (Fig. 31).

The following combination of characters distinguishes this species from other congeners: body narrow, subparallel and brown, with pronotum, elytra and legs lighter, antennae yellowish, surface of forebody moderately glossy and with dense microsculpture, and spermatheca short and S-shaped.

###### Distribution.

This Nearctic species is known only from the type localities in the Yukon Territory.

###### Bionomics.

Adults were collected from May to September from soil and organic litter.

###### Comments.

This species may be easily distinguished by the unique shape of the spermatheca.

**Figures 32–43.**
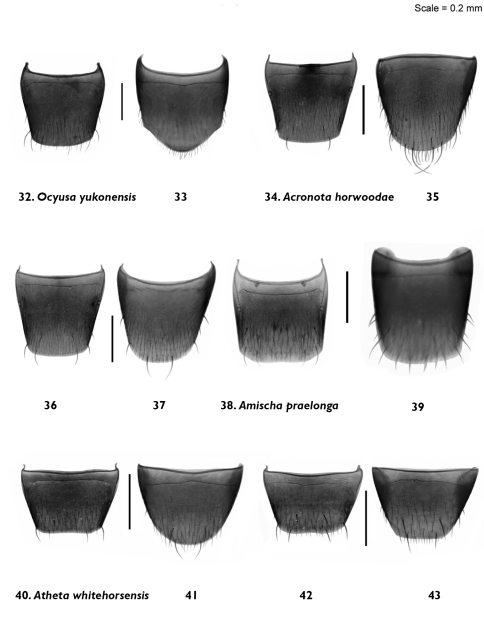
Male and female tergite and sternite 8: *Ocyusa yukonensis* Klimaszewski & Godin, sp. n. **32, 33** male; *Acrotona horwoodae* Klimaszewski & Godin, sp. n. **34, 35**, male **36, 37**, female *Amischa praelonga* (Casey) **38, 39** female; *Atheta (Datomicra) whitehorsensis* Klimaszewski & Godin, sp. n. **40, 41** male **42, 43** female.

**Figures 44–55.**
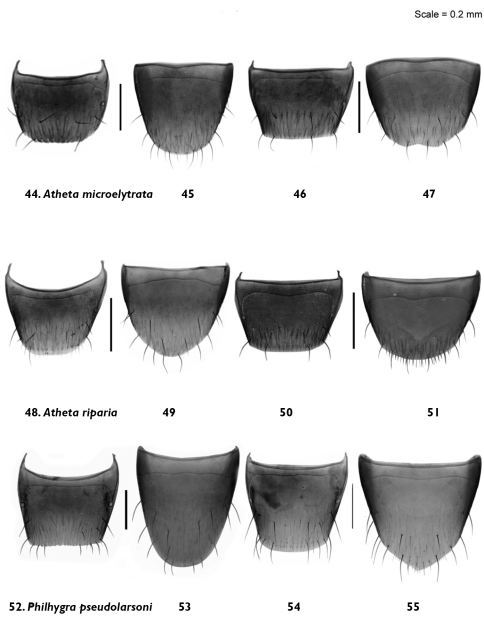
Male and female terite and sternite 8: *Atheta (Microdota) microelytrata* Klimaszewski & Godin, sp. n. **44, 45** male **46, 47** female; *Atheta (Microdota) riparia* Klimaszewski & Godin, sp. n. **48, 49** male **50, 51** female; *Philhygra pseudolarsoni* Klimaszewski & Godin, sp. n. **52, 53**, male **54, 55** female.

**Figures 56–63.**
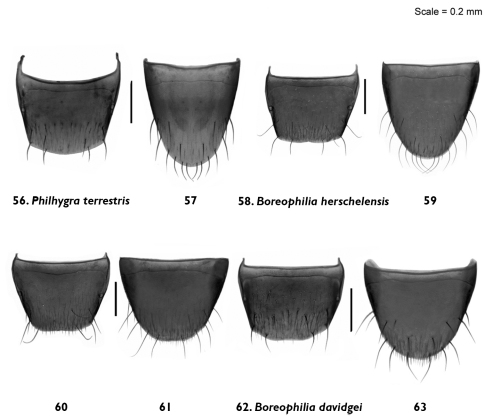
Male and female tergite and sternite 8: *Philhygra terrestris* Klimaszewski & Godin, sp. n. **56, 57** male; *Boreophilia herschelensis* Klimaszewski & Godin, p. n. **58, 59** male**60, 61** female; *Boreophilia davidgei* Klimaszewski & Godin, sp. n. **62, 63** female.

## Supplementary Material

XML Treatment for Calodera parviceps

XML Treatment for Gnathusa eva

XML Treatment for Gnathusa tenuicornis

XML Treatment for Ocyusa yukonensis

XML Treatment for Oxypoda canadensis

XML Treatment for Oxypoda operta

XML Treatment for Brachyusa helenae

XML Treatment for Gyrophaena criddlei

XML Treatment for Acrotona horwoodae

XML Treatment for Strigota ambigua

XML Treatment for Amischa praelonga

XML Treatment for Atheta (Dimetrota) terranovae

XML Treatment for Atheta (Dimetrota) fanatica

XML Treatment for Atheta (Dimetrota) whitehorsensis

XML Treatment for Atheta (Microdota) platonoffi

XML Treatment for Atheta (Microdota) pratensis

XML Treatment for Atheta (Microdota) microelytrata

XML Treatment for Atheta (Microdota) riparia

XML Treatment for Dinaraea angustula

XML Treatment for Lypoglossa franclemonti

XML Treatment for Philhygra pseudolarsoni

XML Treatment for Philhygra sinuipennis

XML Treatment for Philhygra leechi

XML Treatment for Philhygra terrestris

XML Treatment for Philhygra jarmilae

XML Treatment for Boreophilia herschelensis

XML Treatment for Boreophilia davidgei
